# Electrochemical Investigation of the Corrosion of Different Microstructural Phases of X65 Pipeline Steel under Saturated Carbon Dioxide Conditions

**DOI:** 10.3390/ma8052635

**Published:** 2015-05-14

**Authors:** Yuanfeng Yang, Gaurav R. Joshi, Robert Akid

**Affiliations:** School of Materials, the University of Manchester, Manchester M13 9PL, UK; E-Mail: yuanfeng.yang@manchester.ac.uk (Y.Y.); gaurav.joshi-2@postgrad.manchester.ac.uk (G.R.J.)

**Keywords:** X65 pipeline steel, corrosion, carbon dioxide, microstructure, weld, SEM, GIXRD, chukanovite, siderite

## Abstract

The aim of this research was to investigate the influence of metallurgy on the corrosion behaviour of separate weld zone (WZ) and parent plate (PP) regions of X65 pipeline steel in a solution of deionised water saturated with CO_2_, at two different temperatures (55 °C and 80 °C) and at initial pH~4.0. In addition, a non-electrochemical immersion experiment was also performed at 80 °C in CO_2_, on a sample portion of X65 pipeline containing part of a weld section, together with adjacent heat affected zones (HAZ) and parent material. Electrochemical impedance spectroscopy (EIS) was used to evaluate the corrosion behaviour of the separate weld and parent plate samples. This study seeks to understand the significance of the different microstructures within the different zones of the welded X65 pipe in CO_2_ environments on corrosion performance; with particular attention given to the formation of surface scales; and their composition/significance. The results obtained from grazing incidence X-ray diffraction (GIXRD) measurements suggest that, post immersion, the parent plate substrate is scale free, with only features arising from ferrite (α-Fe) and cementite (Fe_3_C) apparent. In contrast, at 80 °C, GIXRD from the weld zone substrate, and weld zone/heat affected zone of the non-electrochemical sample indicates the presence of siderite (FeCO_3_) and chukanovite (Fe_2_CO_3_(OH)_2_) phases. Scanning Electron Microscopy (SEM) on this surface confirmed the presence of characteristic discrete cube-shaped crystallites of siderite together with plate-like clusters of chukanovite.

## 1. Introduction

Dissolved carbon dioxide (CO_2_) in oilfield brines, which accompany extracted oil and gas, causes the internal “sweet” corrosion of steel pipelines. In addition to inducing uniform corrosion, there have been numerous cases of serious localised CO_2_ attack—particularly evident in scale or deposit regions [1]. Even though CO_2_ corrosion product scales are known to often substantially reduce steel corrosion by offering a physical/diffusion barrier on the steel surface, and thereby inhibiting further corrosion [2,3], localised attack can proceed in regions where the surface scales do not offer sufficient protection [4]. Understanding the formation, chemistry and role of CO_2_-induced corrosion scales and surface films in oilfield environments thus remains of considerable interest.

Concerning CO_2_ oilfield corrosion, one specific topic of interest in recent years has been the preferential weld corrosion of carbon steels [5,6,7,8]. Olsen and co-workers suggest that weld failures are a combination of the corrosion susceptibility of the weld and the film forming properties of the CO_2_ saturated environment [9]. In considering CO_2_ corrosion in weld regions, studies have shown that there is a strong correlation between susceptibility to corrosion and weld composition. For example, the use of nickel containing weld consumables during the joining process can lead to preferential corrosion along the weld segment in sweet brines, with significantly less corrosion in welds that were produced using filler metals having no deliberate alloying [5,6,10]. CO_2_ corrosion scales are suspected to form in the initiation stages of weld corrosion [11,12]. Crolet and co-workers highlighted that the nature of the initial phases can determine whether or not the subsequent layer would be protective [13]. Furthermore, corrosion attack may proceed preferentially along the heat-affected zones (HAZ) if the weld region has been protectively covered by scale [9].

To date, there have only been a few studies reporting CO_2_ corrosion/scaling phenomena on low carbon steels in lower pH CO_2_ environments—Particularly with the emphasis on comparing parent-plate material (PP) with weld zones (WZ) [3,6,9,14,15]. On this basis, we report here on the results of electrochemical measurements made on individual parent plate and weld regions of X65 pipeline steel at temperatures of 55 °C and 80 °C. In addition, we report on a non-electrochemical immersion experiment performed on a single sample consisting of weld portion together with HAZ and PP regions, at a temperature of 80 °C, in unstirred CO_2_ saturated deionised water at an initial pH~4.0. The dissolved oxygen level of the test solution was maintained below 10 parts per billion by volume (ppbv) in order to simulate those near-anaerobic conditions usually found within pipelines. Surface corrosion products (scales) have been investigated using scanning electron microscopy (SEM) and grazing incidence X-ray diffraction (GIXRD) techniques.

## 2. Experimental Section

### 2.1. Materials

**Figure 1:**
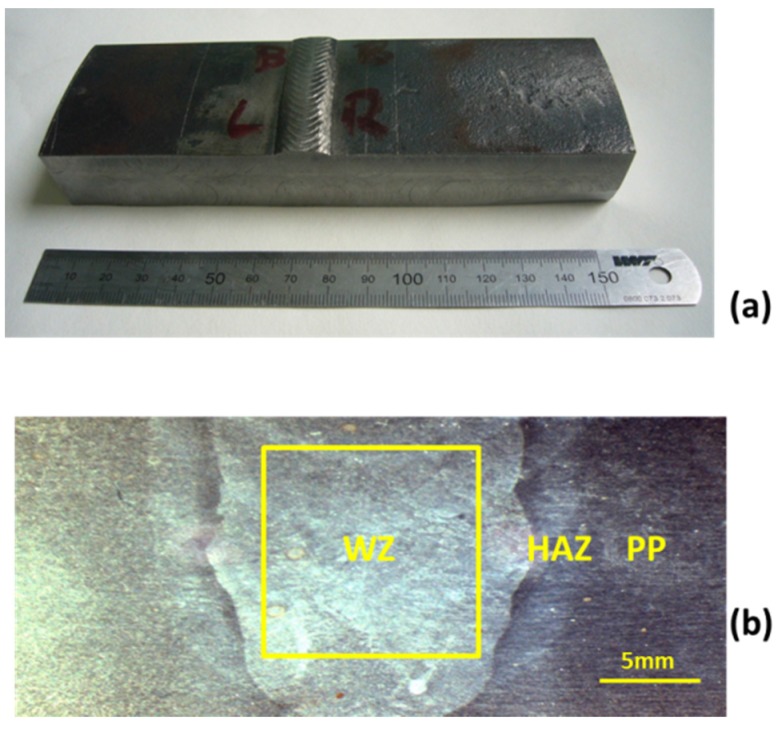
(**a**) Photograph of original portion of X65 pipeline in the “as received” condition; (**b**) Optical micrograph of electropolished cross section of small region of sample shown in (**a**), illustrating part of the Weld Zone (WZ), Heat Affected Zone (HAZ), and Parent Plate (PP). Sample electropolished in 8% perchloric-acetic reagent, at 60 V for 5 s. Yellow box highlighted in weld zone illustrates dimensions of sample that was selected to use in subsequent electrochemical testing.

The original portion of X65 pipeline utilized for the initial set of experiments is shown in Figure 1a, which is illustrated in the “as received” condition. The sample was taken from a commercial pipeline riser, and had been quenched and tempered according to standard industry practice. During production, the pipeline had been welded using gas metal arc welding (GMAW) with a nickel-alloyed carbon steel filler wire. The composition of X65 pipelines steel (UNI EN 10204) parent plate and weld zone filler wire were obtained by x-ray fluorescence spectroscopy and are given in Table 1. Note the difference in nickel content between the PP steel and the composition of the WZ filler. For SEM examination using backscattered electron (BSE) imaging, a water jet cutting system was used to initially cut a region from the original pipeline portion that included part of the weld zone, heat affected zone and parent plate. This sample was polished down to 1/4 µm using diamond paste, then electropolished in 8% perchloric-acetic reagent, at 60 V for 5 s. Figure 1b shows an optical micrograph of the prepared surface of this sample, prior to SEM examination, showing clearly the 3 zones (WZ, HAZ and PP) within the welded pipeline.

**Table 1:** X65 Steel and weld consumable composition (wt%).

Material	C	Mn	Ni	Cr	Mo	Si	Al	Cu	V	P	S	Fe
Parent Plate	0.08	1.08	0.037	0.07	0.13	0.28	0.037	0.16	0.06	0.01	0.001	Balance
Weld Zone filler	0.07	1.46	0.91	0.01	0.01	0.67	0.003	0.11	0.001	0.007	0.009	Balance

**Figure 2:**
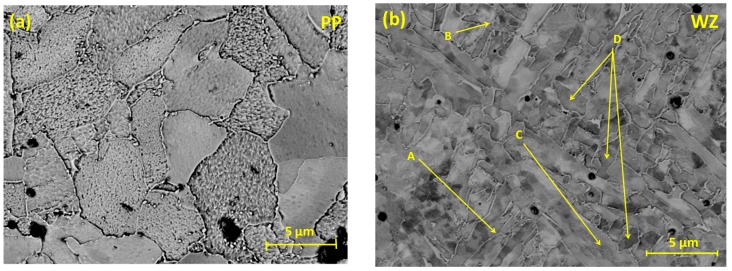
SEM micrographs in BSE mode, of electropolished parent plate and weld regions showing differences in grain morphology: (**a**) Parent Plate (PP) and (**b**) Weld Zone (WZ).

Figure 2 shows BSE images obtained using SEM of the X65 parent and weld regions, clearly illustrating the variation in microstructure between the weld zone and parent plate. The microstructure of the parent plate consists primarily of polygonal ferrite grains (Figure 2a). The microstructure of the weld zone consists of finer grains of bainite (A), acicular ferrite (B), grain boundary ferrite (C), and polygonal ferrite (D) (Figure 2b).

For the electrochemical immersion experiments, samples from both the weld zone and parent plate regions, having dimensions 1.0 cm × 1.0 cm × 1.0 cm, were also cut using a high pressure water jet cutting system. The yellow box highlighted in Figure 1b illustrates the location of the weld zone sample, which was cut from the sample discussed above, whilst the parent plate sample was cut from a site at least 5.0 cm from the weld region, to obviate any effects of heating during welding of the pipeline. The 1.0 cm^2^ area samples (WZ and PP) were spot-welded using Cu–Sn wire, to give an electrical connection, and cold mounted in epoxy resin. Exposed sample surfaces were then re-prepared by polishing down to a 5-μm finish using 4000 grade abrasive paper, cleaned with acetone and stored under a nitrogen atmosphere within a glove box before immersion in the CO_2_ saturated solution.

**Figure 3:**
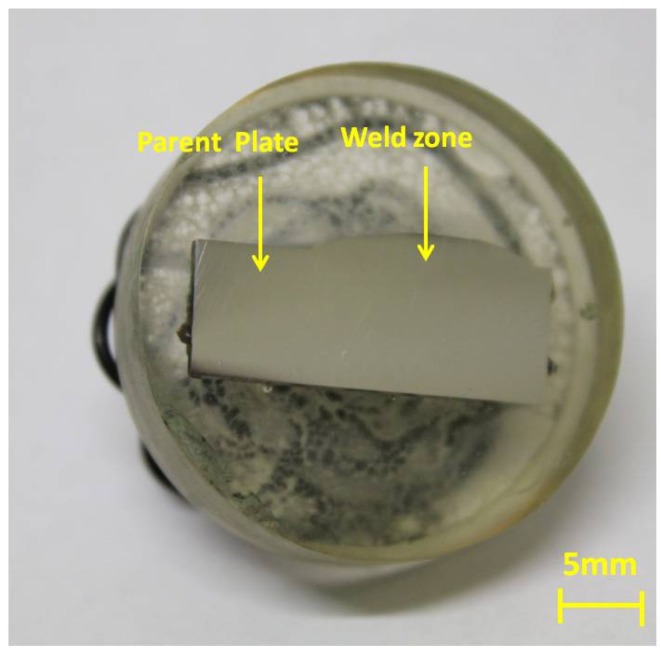
Photograph of single “multi-zone” sample consisting of weld portion together with HAZ and PP before immersion.

An additional sample was also prepared from the original portion of X65 pipeline (shown in Figure 1a), using a high pressure water jet cutting facility, but consisting of a single “multi-zone” sample of weld together with HAZ and PP. This specimen is shown in Figure 3.

### 2.2. Experimental Procedure

The individually prepared 1.0 cm^2^ area separate samples from both WZ and PP were used for the electrochemical corrosion experiments. In addition, a set of non-electrochemical experiments, consisting only of immersion tests, was also carried out using the “multi-zone” sample. All experiments, including the “multi-zone” sample, were performed in a jacketed glass electrochemical cell containing 1 L CO_2_—Saturated deionised water, at initial pH~4 (all pH values determined using a calibrated Hanna instruments pH meter) and all within a N_2_-purged acrylic glovebox. Note that the conductivity of the deionised water was 3 μs/cm and increased to 280 μs/cm when fully saturated with CO_2_.

The electrolyte temperature was maintained by circulating heated water from a water bath, through the surrounding jacket cell. The electrolyte was previously purged with high purity CO_2_ gas (BOC, Ashton-under-Lyne, Lancashire, UK, 99.995%) for 18–24 h prior to commencing the experiment, to ensure low dissolved oxygen concentration, which was measured using a commercial electrochemical sensor. Once the oxygen concentration was determined to be less than 10 ppb, the CO_2_ gas flow was changed to pass over the solution surface (blanket flow), maintaining a constant pressure of 1 bar. This ensured that the solution remained static (natural convention flow) for the experiment duration; and the CO_2_—Water equilibrium was maintained. Any water loss was considered to be less than 5%.

Electrochemical measurements were performed using either an X65 parent plate or weld zone sample as the working electrode (WE) with a platinum-mesh counter electrode (CE) and an Ag/AgCl reference electrode (RE). A polymer gel salt bridge was used to connect the RE to the test solution in order to minimise any contamination from the reference electrode reservoir solution (3.5 M KCl). All experiments were of 72 h duration. Throughout almost all of the immersion period, the WE was maintained at open circuit potential (OCP), except for hourly electrochemical impedance spectroscopy (EIS) measurements, involving sample polarisation of ±10 mV *vs.* OCP, over a frequency range of 10 kHz–50 mHz. EIS data was acquired using a Solartron Potentiostat 2100 A (Farnborough, Hampshire, UK). After the experiment, the WE samples were stored in a vacuum dessicator to avoid surface oxidation. The samples were then subjected to GIXRD and surface SEM analysis. All of the GIXRD measurements used a CuKα source and were carried out at a 3° incidence angle using a Philips XPERT XRD diffractometer (PANalytical Ltd., Cambridge, UK) in the 2θ range from 5° to 85°. A FEI XL-30 FEGSEM (Hillsboro, OR, USA) was used for all SEM examination throughout this work.

### 2.3. Interpreting EIS for Obtaining Corrosion Rates of X-65 Parent/Weld Samples

Corrosion rate (*i*_corr_) was estimated by extracting the observed *R*_ct_ from the EIS spectra and applying the *B* parameter [3,16] in order to calculate corrosion rate. Equation (2) was used to estimate the corrosion rate of the steel over the 72 h immersion period.

For calculating the corrosion rates, we applied Faraday’s law and the Stern-Geary relation, using the Stern-Geary coefficient *B* = 26 mV:

(1) $$R_{p}=\frac{B}{i_{\text{corr}}}$$

(2) $$CR(\text{mm/y})=\frac{i_{\text{corr}}A_{w}\times 10\times 3.15\times 10^{7}}{nF\rho }$$

where: *A*_w_ = 56 g/mol of iron (Fe) is assumed for carbon steel. *F* = Faraday’s Constant (96,500 C/mol). *n* = 2 and ρ = 7.87 g/cm^3^ for carbon steel.

## 3. Results and Discussion

### 3.1. Corrosion Rates of X65 Parent and Weld Samples

**Figure 4:**
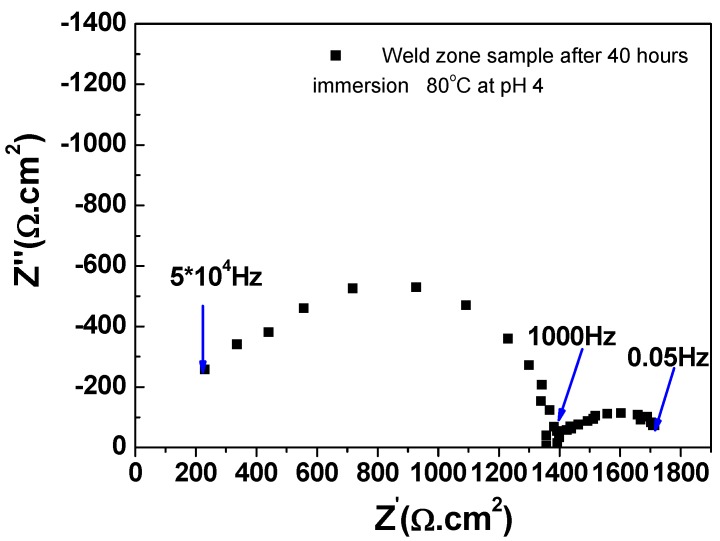
An example Nyquist plot of X65 weld zone sample after 40 h immersion in CO_2_ saturated deionised water at 80 °C and initial pH~4.0.

Figure 4 shows a typical EIS Nyquist plot, acquired in this study. The spectrum consists of two semicircles; one large semicircle at high frequencies and one depressed semicircle at lower frequencies. We interpret the response at higher frequencies to be associated with the low conductivity of the solution leading to a significant solution resistance between the RE and WE. The profile exhibited at lower frequencies is associated with the steel corrosion process [17]. It should be noted that the diameter of the higher frequency semicircle (50 kHz–1 kHz) remained constant throughout the 72 h experiment.

**Figure 5:**
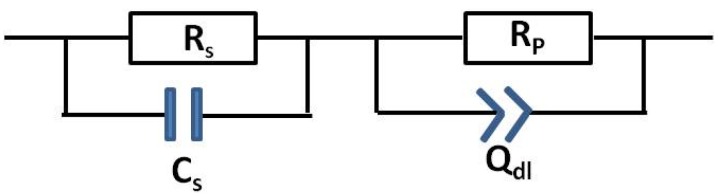
Equivalent circuit for samples immersed in CO_2_ saturated deionised water.

In order to extract useful information regarding the corrosion process (such as the corrosion rate), the EIS data was analysed using the equivalent circuit model shown in Figure 5. Here, *R*_S_ is solution resistance; *C*_S_ is the capacitance associated with the low conductivity solution; *R*_P_ is the polarisation resistance (corrosion); and *Q*_dl_ is a constant phase element (capacitance) associated with the solution/metal interface.

**Figure 6:**
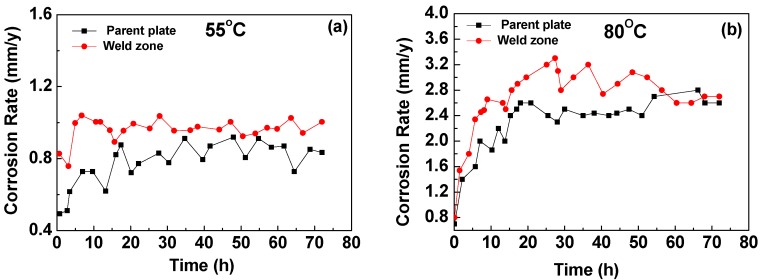
Plots comparing the corrosion rates of X65 parent plate and weld zone samples after 72 h immersion in CO_2_ saturated deionised water; (**a**) 55 °C at initial pH~4.0, (after 72 h, pH was 4.1~4.2 for both samples); (**b**) 80 °C at initial pH~4.0, (after 72 h, parent plate pH was 4.3, weld zone pH was 4.6).

Corrosion rates at 55 °C and 80 °C are plotted in Figure 6a,b respectively. The corrosion rate profiles for the parent plate, in Figure 6a, settled to between 0.7 and 0.9 mm/year after 7–8 h immersion until the experiment finished. For comparison, the corrosion rates of the weld zone sample, after 7–8 h immersion, were between 0.9 and 1.0 mm/year until the experiment finished. It suggests that no significant/protective corrosion scales were formed on the surface in these immersion experiments at 55 °C [17].

Analysis of corrosion rates for the parent plate sample after 7 to 8 h immersion at 80 °C, Figure 6b, shows that the rate settled to between 2 and 2.6 mm/year, while the equivalent corrosion rate for the weld zone sample was around 2.4 to 3.3 mm/year after 7 to 8 h immersion. For both the 55 °C and 80 °C conditions, the corrosion rates for the weld zone sample were higher than the corrosion rates obtained for the parent plate sample. It was observed that in the weld zone sample at 80 °C (Figure 6b), the corrosion rate had slightly dropped after 28 h immersion from 3.3 to 2.8 mm/year. The corrosion rates observed for the parent plate sample remained quite constant at both 55 °C and 80 °C.

Olsen and co-workers [9] studied uncoupled X52 parent plate steel and 1% Ni-weld zone corrosion in CO_2_—Saturated 0.06 M NaCl solutions at 60 °C and pH~5–6. They reported that: “typically, the weld metal shows preferential corrosion in the early part of the test and then falls. Some segments of the weldment samples begin to form a protective scale on the surface”. In this study, whilst there is a drop in corrosion rate from 3.3 drop to 2.8 mm/year, this change (≈15%) is within the scatter of the data, and, therefore, without extending the duration of exposure, it is difficult to conclude that the scale provides any long-term protection.

### 3.2. Surface Analysis

**Figure 7:**
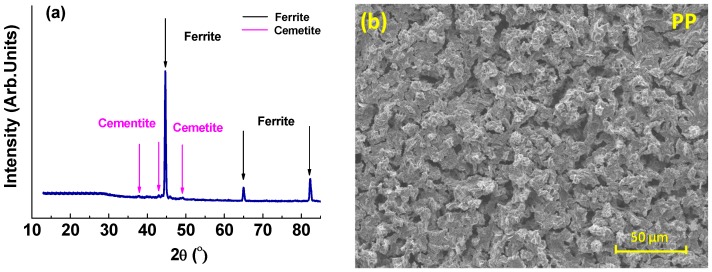
Analysis of parent plate sample after 72 h immersion at 55 °C and at initial pH~4.0 (Note: after 72 h the pH was recorded at 4.1): (**a**) GIXRD spectrum and (**b**) Secondary-electron SEM image.

**Figure 8:**
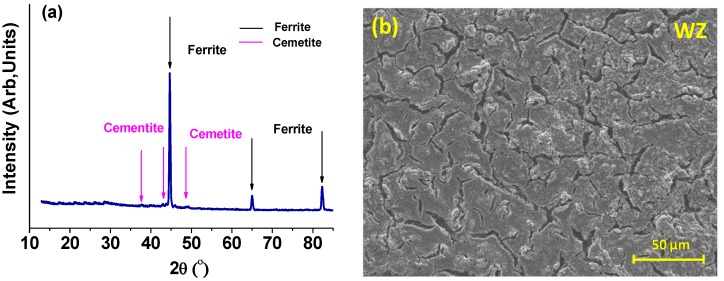
Analysis of weld zone sample after 72 h of immersion at 55 °C and at initial pH~4.0 (Note: after 72 h, pH was 4.1) (**a**) GIXRD spectrum and (**b**) Secondary-electron SEM image.

**Figure 9:**
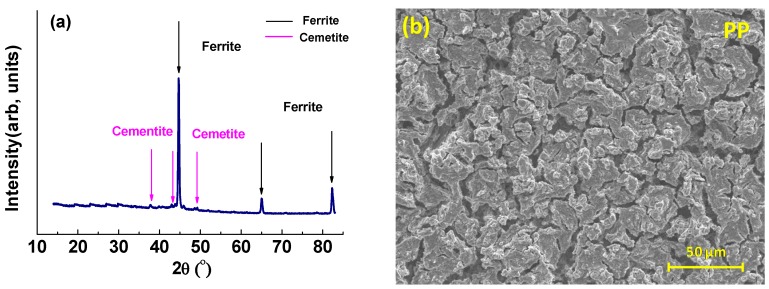
Analysis of parent plate sample after 72 h of immersion at 80 °C and at initial pH~4.0 (Note: after 72 h, pH was 4.3): (**a**) GIXRD spectrum and (**b**) Secondary-electron SEM image.

**Figure 10:**
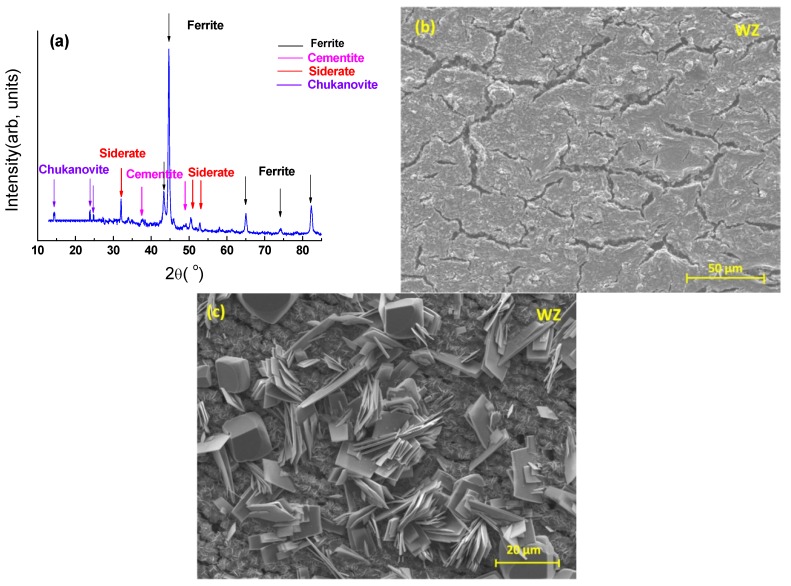
Analysis of weld zone sample after 72 h of immersion at 80 °C and at initial pH~4.0 (Note: after 72 h, pH was 4.6): (**a**) GIXRD spectrum; (**b**) Secondary-electron SEM image of weld zone (most of the sample surface showed this morphology); and (**c**) Secondary-electron SEM image of different region of weld zone (to Figure 10b), (a small percentage of the sample surface showed this morphology).

The results of post-immersion analysis of the X65 parent plate (PP) and weld zone (WZ) samples at 55 °C and 80 °C using GIXRD and SEM are shown in Figure 7, Figure 8, Figure 9 and Figure 10.

For both the parent plate and weld zone samples immersed at 55 °C and for the parent plate sample immersed at 80 °C, only the diffraction peaks assigned to ferrite and cementite were observed (see Figure 7a, Figure 8a and Figure 9a). In agreement with these results, SEM images also show no evidence of surface scaling, rather they show typical surface morphologies consistent with uniform CO_2_ corrosion [18]. From these observations, along with the measured corrosion rates (see Figure 6a,b), it is suggested that scale-free corrosion took place on these substrates.

In contrast, GIXRD data acquired from the weld zone sample immersed at a solution temperature of 80 °C, as shown in Figure 10a, revealed the presence of chukanovite (Fe_2_CO_3_(OH)_2_), siderite (FeCO_3_), ferrite (α-Fe) and cementite. In Figure 10b, there is no obvious scale after 72 h exposure to a CO_2_ environment. The surface morphology shown in this figure was typical of 90% of the sample surface area examined. However, in contrast, see Figure 10c the surface morphology observed, typical of only about 10% of the surface sample area, consisted of small cubic and discrete plate-like crystals. The presence of chukanovite and siderite diffraction peaks obtained from GIXRD are consistent with the SEM images in Figure 10c. The plate-like crystals are essentially identical in morphology to those assigned to chukanovite by other workers [19,20]. Similarly, the cuboid-shaped crystals are characteristic of siderite [16].

**Figure 11:**
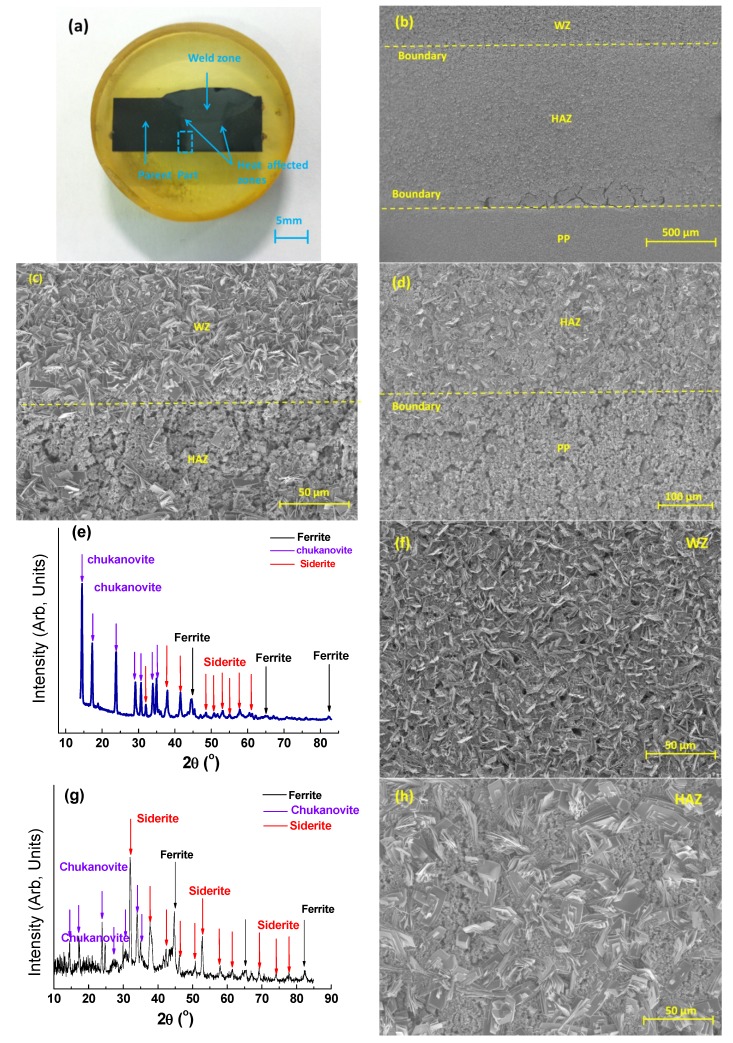

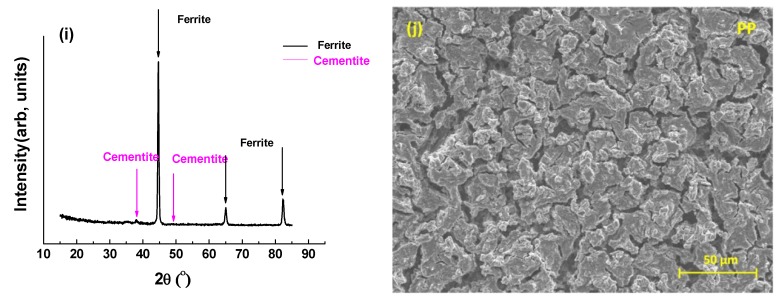
Analysis of “multi-zone” sample after 72 h immersion in CO_2_ saturated deionized water at 80 °C, with initial pH~4.0 (Note: after 72 h the pH was 5.2): (**a**) Optical photograph of whole sample after 72 h immersion, the weld portion together with HAZ and PP are clearly visible. Note: Figure 3 illustrates same sample prior to immersion; (**b**) Secondary electron SEM image of area highlighted in blue rectangle in (**a**), Note: all SEM images in this Figure are orientated 90° clockwise; (**c**) Secondary electron SEM image of boundary region between parent plate (PP) and heat affected zone (HAZ), showing clear distinction in the morphology of the surface deposits in the two zones; (**d**) Secondary electron SEM image of the boundary between the heat-affected zone (HAZ) and the weld zone (WZ), also showing a clear distinction in surface morphology; (**e**) GIXRD spectrum from weld zone (WZ) only; (**f**) Secondary electron SEM image of weld zone only, image corresponds to spectrum given in (**e**); (**g**) GIXRD spectrum of heat affected zone (HAZ) only; (**h**) Secondary electron SEM image of heat affected zone (HAZ) only, image corresponds to spectrum given in (**g**); (**i**) GIXRD spectrum of parent plate (PP) region only; (**j**) Secondary electron SEM image of parent plate (PP) region only, image corresponds to spectrum given in (**i**).

Analysis of the single “multi-zone” sample, consisting of weld portion together with HAZ and PP, immersed at 80 °C, is given in Figure 11a. This is supported by GIXRD and SEM data of the individual regions of “multi-zone” sample, as shown in Figure 11b–j.

Figure 11 shows the results of the SEM and GIXRD analysis of the “multi-zone” sample, and illustrates the significant differences in surface morphology and deposit composition, corresponding to the different sample regions, that occur after 72 h immersion. The three different zones are clearly visible in the optical photograph presented in Figure 11a, and these differences were visible with the naked eye, even after 7 to 8 h immersion. After 72 h, the differences in morphology of the surface deposits on the three zones were clearly discernable in the low magnification secondary electron image shown in Figure 11b. Figure 11c,d present higher magnification secondary electron SEM images of the WZ to HAZ and HAZ to PP interfaces respectively. These images illustrate very clearly the abrupt changes in surface deposit morphology that occur at both zone boundaries.

The individual GIXRD spectra and corresponding secondary electron SEM images of the WZ, HAZ and PP zones are given in Figure 11e–j. For the WZ region (Figure 11e), the GIXRD spectrum of the surface revealed the presence of chukanovite (Fe_2_CO_3_(OH)_2_), siderite (FeCO_3_) and ferrite (α-Fe). The intensity of the chukanovite peaks relative to the ferrite and siderite peaks was clearly far greater, indicating that there might have been a significant surface coverage and presence of this phase. SEM analysis confirmed this particular hypothesis (Figure 11f) showing evidence of typical plate-like chukanovite crystals. On the HAZ region (Figure 11g), the GIXRD spectrum of the surface deposit revealed the presence of chukanovite (Fe_2_CO_3_(OH)_2_), siderite (FeCO_3_) and ferrite (α-Fe), which corresponds to the characteristic surface deposit morphologies observed in the corresponding SEM image (Figure 11h), with evidence of typical cuboidal shaped siderite surrounded by characteristic clusters of plate-like chukanovite crystals. On the PP (Figure 11i), the GIXRD spectrum of the surface deposit revealed only the presence of cementite and ferrite; this was confirmed by SEM observation (Figure 11j).

It is proposed that an even greater quantity of iron will have dissolved from the “multi-zone” sample consisting of a weld portion together with HAZ and PP than the other individual PP and WZ samples, since significant iron-rich corrosion scales were deposited on WZ. After 72 h, the pH attained by the “multi-zone” sample was 5.2, which was the highest pH values recorded across all of the tests conducted. From reports in the literature, scale formation is not usually expected at pH values below 5.5 [18]. At pH conditions less than this, chukanovite and siderite are not expected to precipitate out of solution. Such scales formation has not often reported on steel surfaces in the open literature, particular in fairly acidic bulk solutions. More commonly, chukanovite is detected as a minority phase that is secondary to siderite [14,21]. Furthermore, bulk acidic solutions would likely encourage the dissolution of chukanovite in the first instance, followed by dissolution of siderite [22].

In the last decade, chukanovite has been discussed in great detail in the fields of iron archaeology and long-term nuclear storage, typically being found under anaerobic environments. A study by Saheb on archaeological iron, has shown that in carbonate-rich, anaerobic conditions, Fe_2_CO_3_(OH)_2_ forms first on iron metal surfaces followed by FeCO_3_ [21]. There is general agreement in the literature that this compound forms under near neutral-alkaline conditions (>pH 6) [23,24]. By varying the concentration ratios of FeCl_2_ (aq), NaOH (aq) and Na_2_CO_3_ (aq) in Ar-deaerated water, and characterizing the precipitates formed (using FT-IR), Remazeilles *et al* have indicated that the preferential formation of Fe_2_(OH)_2_CO_3_ is reliant upon the ratio of ratio of [Fe^2+^(aq)] to [OH^−^(aq)] and [Fe^2+^(aq)] to [CO_3_^2−^(aq)] [25]. Experimental work by Han *et al*, concerned with interfacial pH measurements at a carbon steel mesh corroding in CO_2_-saturated solutions at 80 °C, appears to suggest that even if the bulk solution pH is fairly acidic (~4), the pH at the metal/solution interface is likely less so (~6)—*i.e*., higher local [OH^−^(aq)] [26]. A recent report by Refait *et al* has suggested that the formation of either chukanovite, siderite or other compounds (carbonated green rust or magnetite) at a carbon steel surface in anaerobic carbonate-rich solutions must be controlled primarily by the [Fe^2+^(aq)] to [OH^-^(aq)] and [Fe^2+^(aq)] to [CO_3_^2−^(aq)] ratios at the metal/solution interface [27]. In particular, if the relative molar ratio of [Fe^2+^(aq)]:[OH^−^(aq)] is approximately 1 and [Fe^2+^(aq)]:[CO_3_^2−^(aq)] is approximately 2, thermodynamically stable chukanovite formation should dominate in preference to Fe(OH)_2_ or FeCO_3_. In such a case, the nature of the surface film should be determined by the kinetics of iron dissolution.

From our electrochemical observations, over the first 30 h the weld zone at 80 °C demonstrated a higher corrosion rate than the parent plate (see Figure 6b). This initial period of “accelerated corrosion” leads to the production of a high local surface concentration of Fe^2+^. Therefore, on the weld surface, the observed initial severe corrosion rate perhaps equated to the generation of a high interfacial ratio of Fe^2+^ with respect to CO_3_^2−^. This coupled with a probable equivalence of Fe^2+^ to OH− (from the relatively higher interface pH) may have led to the formation of stable chukanovite.

The observed chukanovite on the weld zone may have acted as a pre-cursor to siderite formation [14], but this would need to be confirmed with longer immersion experiments. Finally, it remains in debate whether or not it was the initial high corrosion rate that was critical in developing a chukanovite-rich surface scale, or whether the weld microstructure and composition played an important role.

## 4. Conclusions

- 72 h electrochemical corrosion immersion experiments on X65 parent and weld regions were performed in CO_2_-saturated deionised water at 55 °C and 80 °C at an initial pH~4. After 72 h immersion, the measured corrosion rates were related to surface deposit characterisation using GIXRD and SEM.
- At both temperatures, the separate weld zone initially corroded at a higher rate than the parent plate.
- At 55 °C, GIXRD offered no evidence of scaling, and no crystallites could be observed from SEM images of both X65 parent and weld regions. At 80 °C, similar observations held for the X65 parent plate. The sample surfaces exhibit uniform corrosion in these environments.
- The X65 weld sample, at 80 °C, corroded more rapidly during the initial 30 h, but then fell to a lower corrosion rate and remained relatively stable for the remaining period of immersion. The surface was covered with chukanovite (Fe_2_CO_3_(OH)_2_), along with some siderite (FeCO_3_), which was confirmed from GIXRD and SEM studies, and supported by published literature.
- A non-electrochemical immersion experiment was performed on a single “multi-zone” sample consisting of a weld portion together with HAZ and PP zones at 80 °C. A simple optical micrograph of the whole sample surface clearly distinguished the three different zones. From GIXRD and SEM results, the surface of the WZ/HAZ showed extensive presence of chukanovite (Fe_2_CO_3_(OH)_2_), along with some siderite (FeCO_3_), whilst very little scaling was observed on the PP sample surface.
- Understanding the impact of weld microstructure and composition on the initial higher rate of corrosion and possible subsequent chukanovite formation, along with the exact role of this corrosion scale, remains a topic of further research.
