# Correction: Electrochemical Investigation of the Corrosion of Different Microstructural Phases of X65 Pipeline Steel under Saturated Carbon Dioxide Conditions. *Materials* 2015, *8*, 2635–2649

**DOI:** 10.3390/ma8125485

**Published:** 2015-12-11

**Authors:** Yuanfeng Yang, Gaurav R. Joshi, Robert Akid

**Affiliations:** School of Materials, The University of Manchester, Oxford Road, Manchester M13 9PL, UK; Yuanfeng.Yang@manchester.ac.uk (Y.Y.); gaurav.joshi-2@postgrad.manchester.ac.uk (G.R.J.)

In the published manuscript “Electrochemical Investigation of the Corrosion of Different Microstructural Phases of X65 Pipeline Steel under Saturated Carbon Dioxide Conditions. *Materials* 2015, *8*, 2635–2649 [1]”, we acknowledge that a contributor (Gaurav R. Joshi) had been omitted from the author list. Alterations are therefore requested to the author list, “Corresponding Author”, “Acknowledgements” and “Author Contributions”. Furthermore, we have since noted that in three places within the article, inappropriate references have been used. We apologize for the inconvenience this will have caused. The following changes to the text have been made:

Page 2646, Section 3.2, “Surface Analysis”:

“More commonly, chukanovite is detected as the minority phase, and usually secondary to siderite [14,21,22].”

Is replaced with “More commonly, chukanovite is detected as a minority phase that is secondary to siderite [14,21].”

Page 2646, Section 3.2, “Surface Analysis”:

“Furthermore, acidic solutions would be considered to enhance the dissolution of chukanovite in the first instance, followed by the dissolution of siderite [23,24].”

Is replaced with “Furthermore, bulk acidic solutions would likely encourage the dissolution of chukanovite in the first instance, followed by dissolution of siderite [22].”

Page 2646, Section 3.2, “Surface Analysis”:

“There is general agreement in the literature that this compound forms under slightly alkaline conditions (>pH 6) [25,26].”

Is replaced with “There is general agreement in the literature that this compound forms under near neutral-alkaline conditions (>pH 6) [23,24].”

Page 2646, Section 3.2, “Surface Analysis”:

“Later work by Remazeilles has suggested that the preferential formation of Fe_2_(OH)_2_CO_3_ depended upon the ratio of [Fe^2+^] to [OH^−^] and [Fe^2+^] to [CO_3_^2−^] in his deaerated FeCl_2_/NaOH/Na_2_CO_3_ solutions [27].”

Is replaced with “By varying the concentration ratios of FeCl_2_ (aq), NaOH (aq) and Na_2_CO_3_ (aq) in Ar-deaerated water, and characterizing the precipitates formed (using FT-IR), Remazeilles *et al.* have indicated that the preferential formation of Fe_2_(OH)_2_CO_3_ is reliant upon the ratio of ratio of [Fe^2+^(aq)] to [OH^−^(aq)] and [Fe^2+^(aq)] to [CO_3_^2−^(aq)] [25].”

Page 2646, Section 3.2, “Surface Analysis”:

“Subsequently, experimental work by Han reported that, even if the bulk solution pH is low (~4), local pH measurements on actively corroding steel surfaces in CO_2_ saturated environments at 80 °C showed that the pH at the interface is more alkaline (~6) [28].”

Is replaced with “Experimental work by Han *et al.*, concerned with interfacial pH measurements at a carbon steel mesh corroding in CO_2_-saturated solutions at 80 °C, appears to suggest that even if the bulk solution pH is fairly acidic (~4), the pH at the metal/solution interface is likely less so (~6)—*i.e*., higher local [OH^−^(aq)] [26].”

Page 2646, Section 3.2, “Surface Analysis”:

“In particular, if the relative molar ratio of [Fe^2+^]:[OH^−^] is approximately 1 and [Fe^2+^]:[CO_3_^2−^] is approximately 2, thermodynamically stable chukanovite formation dominates in preference to Fe(OH)_2_ or FeCO_3_. A very recent report by Refait suggested that the formation of either chukanovite, siderite or other compounds (carbonated green rust or magnetite) at a steel surface under anaerobic carbonate-rich solutions must be controlled primarily by the aforementioned concentration ratios at the metal/solution interface [29].”

Is replaced with “A recent report by Refait *et al.* has suggested that the formation of either chukanovite, siderite or other compounds (carbonated green rust or magnetite) at a carbon steel surface in anaerobic carbonate-rich solutions must be controlled primarily by the [Fe^2+^(aq)] to [OH^-^(aq)] and [Fe^2+^(aq)] to [CO_3_^2−^(aq)] ratios at the metal/solution interface [27]. In particular, if the relative molar ratio of [Fe^2+^(aq)]:[OH^−^(aq)] is approximately 1 and [Fe^2+^(aq)]:[CO_3_^2−^(aq)] is approximately 2, thermodynamically stable chukanovite formation should dominate in preference to Fe(OH)_2_ or FeCO_3_.”

Page 2646, Section 3.2, “Surface Analysis”:

“The observed chukanovite observed on the weld zone may have acted as a pre-cursor to siderite formation but this would also need to be confirmed with longer immersion experiments [14,30].”

Is replaced with “The observed chukanovite on the weld zone may have acted as a pre-cursor to siderite formation [14], but this would need to be confirmed with longer immersion experiments.”

Page 2648, “Acknowledgments”:

“The author Yuanfeng Yang wishes to thank Mr G. R. Joshi for assistance with the glove box and experimental set up, and some discussion of the results. Dr Chris Wilkins and Mr Gary Harrison are thanked for their expert contribution with the SEM and XRD analysis.”

Is replaced with “Gaurav R. Joshi acknowledges funding from the EPSRC via the Advanced Metallic Systems CDT. The authors thank Robert Lindsay for his comments during the discussion of the results, and Chris Wilkins and Gary Harrison for their assistance during the acquisition and analysis of the SEM and GIXRD data.” 

Page 2648, “Author Contributions”:

“Yuanfeng Yang planned and conducted all experiments, examined the experimental results, performed all data analysis and wrote the article draft.”

Is replaced with “Yuanfeng Yang planned and conducted all experiments. Yuanfeng Yang and Gaurav R Joshi examined the experimental results, performed data analyses and wrote the article draft together. Robert Akid was involved in the discussions, and proposed revisions to the final draft of the article script.”

The manuscript will be updated and the original remain online at the article webpage.
